# The Association Between Chronic Pain Acceptance and Pain-Related Disability: A Meta-Analysis

**DOI:** 10.1007/s10880-024-10061-1

**Published:** 2024-12-16

**Authors:** Kyle M. White, Emily L. Zale, Emma C. Lape, Joseph W. Ditre

**Affiliations:** 1 Department of Psychology, Syracuse University, 352 Marley Educational Building, 765 Irving Avenue, Syracuse, NY 13244 USA; 2 Department of Psychology, Binghamton University, Binghamton, NY 13902 USA

**Keywords:** Chronic pain, Pain acceptance, Disability, Meta-analysis

## Abstract

Chronic pain acceptance has been identified as a crucial determinant of functioning among individuals living with chronic pain. However, research examining the relationship between chronic pain acceptance and pain-related disability has revealed variability across studies. The aims of this meta-analysis were to synthesize associations between chronic pain acceptance and pain-related disability, and test the moderating influence of pain characteristics, demographic factors, and methodological variables. Searches of PubMed and PsycINFO electronic databases located 24 studies (*N* = 6072) reporting correlations between chronic pain acceptance and pain-related disability. Meta-analytic calculations were performed using random effects models. Results indicated a negative association between chronic pain acceptance and pain-related disability that can be characterized as moderate in magnitude (*r* = −.45) and was stable across pain characteristics and demographic factors. Moderator analyses suggested the association between chronic pain acceptance and pain-related disability may be sensitive to the measures used to assess these constructs. These findings underscore the role of chronic pain acceptance in pain-related disability and reinforce the clinical significance of acceptance-based approaches in enhancing functional outcomes among chronic pain patients.

**Supplementary Information:**

The online version contains supplementary material available at 10.1007/s10880-024-10061-1.

## Introduction

Chronic pain is a prevalent public health concern, affecting an estimated 20.5% of the U.S. adult population each year (Yong et al., 2022). Although many chronically painful conditions are not directly life-threatening, chronic pain places immense strain on individuals, health care systems, and economies (Gaskin & Richard, 2012; Goldberg & McGee, 2011; Phillips, 2009). Indeed, chronic pain is consistently identified as a leading cause of disability worldwide (Hay et al., 2017; Rice et al., 2016; Vos et al., 2020). Pain-related disability describes the functional limitations associated with the experience of pain and includes physical, occupational, recreational, and social domains (Pollard, 1984). Given that measures of pain-related disability only show modest associations with pain intensity (Bahat et al., 2014; Garbi et al., 2014; Grönblad et al., 1993) and have been shown to independently predict depression, employment status, and medication usage (Bean et al., 2014; Jerome & Gross, 1991), pain-related disability has been recognized as a core outcome that should be assessed in all chronic pain clinical trials (Dworkin et al., 2005).

Early psychological interventions for chronic pain emphasized operant-behavioral principles, aiming to mitigate maladaptive pain behaviors via contingency management (Fordyce, 1976). These behavioral approaches were subsequently expanded to include cognition and constructs such as catastrophizing and pain self-efficacy (Turk et al., 1983; Turner & Romano, 2001). Stemming from a functional contextual framework, third-generation behavioral therapies such as Acceptance and Commitment Therapy (ACT) have been successfully applied to pain (Hayes & Duckworth, 2006; Hughes et al., 2017). Within this perspective, no psychological experience is viewed as pathological in and of itself; rather, it is the context in which mental phenomena unfold that can impart deleterious effects (e.g., a context predominated by experiential avoidance; Hayes et al., 2006). Third-generation psychotherapies, therefore, emphasize the improvement of functioning despite the continued presence of pain symptoms, and acceptance is a critical process by which this is achieved (Hayes & Duckworth, 2006).

Chronic pain acceptance has been defined as a psychological state of willingness to experience chronic pain and its sequelae while continuing engagement in valued life activities (Reneman et al., 2010). Although the strength of reported associations varies, previous research has shown that acceptance of chronic pain is a predictor of lower levels of pain-related disability (Kanzler et al., 2019; Nicholas & Asghari, 2006; Sardá et al., 2009). Two qualitative reviews have highlighted the critical influence of acceptance on the functioning of individuals with chronic pain (McCracken & Vowles, 2006; Thompson & McCracken, 2011). Importantly, the research presented in these reviews suggests that chronic pain acceptance is modifiable in the context of treatment. For example, in a heterogeneous sample of 108 chronic pain patients that received acceptance-based behavioral therapy, post-treatment improvements in disability were associated with changes in chronic pain acceptance (McCracken et al., 2005). This finding was later replicated in a larger sample of 252 chronic pain patients receiving the same acceptance-based behavioral therapy, with increased chronic pain acceptance predicting reductions in disability at 3-month follow-up (Vowles et al., 2007). Collectively, these studies underscore the role of chronic pain acceptance in pain-related disability and support the potential clinical utility of approaches that target acceptance.

Despite growing evidence linking acceptance to functional outcomes in chronic pain, we are unaware of any meta-analytic studies that estimated the strength of the relationship between chronic pain acceptance and pain-related disability. The variability in the literature may imply the existence of moderators; however, understanding of these factors remains limited. Therefore, the goals of the current meta-analysis were to quantify the magnitude of the association between chronic pain acceptance and pain-related disability, and examine the influence of potential moderators, including pain characteristics, demographic factors, and methodological variables.

## Method

### Search Procedure

This meta-analysis was conducted in accordance with PRISMA (Preferred Reporting Items for Systematic Reviews and Meta-Analyses; Page et al., 2021) guidelines and was not pre-registered. Eligible studies published prior to February 2023 were identified using PubMed and PsycINFO electronic databases. Keywords, in conjunction with MeSH terms (PubMed) or Subject Headings (PsycINFO), were employed for database searches, encompassing the concepts of *pain*, *acceptance*, and *disability*. The complete search strategy can be found in Supplementary Information. Reference lists of eligible studies were manually searched for additional relevant articles.

### Determination of Outcome Variables

Chronic pain acceptance is assessed via self-report measures, which evaluate a state of willingness to experience chronic pain while persisting with valued life activities (Reneman et al., 2010). The Chronic Pain Acceptance Questionnaire (CPAQ; McCracken et al., 2004) is one such instrument and is composed of two subscales: Pain Willingness and Activity Engagement. Pain Willingness assesses the willingness to have pain present without attempting to eliminate or reduce it (e.g., “I avoid putting myself in situations where my pain might increase” and “I need to concentrate on getting rid of my pain”). Activity Engagement assesses the degree to which individuals participate in life activities despite pain (e.g., “My life is going well, even though I have chronic pain” and “When my pain increases, I can still take care of my responsibilities”).

Pain-related disability is frequently assessed via self-report measures. These instruments can be general, referring to pain without reference to a particular site or type (e.g., Pain Disability Index; Pollard, 1984), or condition-specific (e.g., Quebec Back Pain Disability Scale; Kopec et al., 1996). Self-report pain-related disability measures evaluate functional limitations associated with the experience of pain. A summary of the chronic pain acceptance and pain-related disability measures from which data were derived is presented in Supplementary Information.

Potential moderators were identified a priori and included the specific predictor and criterion measures used, pain characteristics (i.e., type, duration, intensity, and treatment status), demographic factors (i.e., gender and age), and study quality. These moderators were selected because they each address considerations relevant to the association between chronic pain acceptance and pain-related disability. For example, studies have raised concern about the possibility of floor and ceiling effects in pain-related disability measures, which could complicate the interpretation of extreme scores (Brodke et al., 2017; Roland & Fairbank, 2000). Regarding pain characteristics, factors such as duration and intensity have been positively associated with disability (Bean et al., 2014; Duyur Çakıt et al., 2009; Feinstein et al., 2011) and may be more severe/persistent in treatment-seeking samples. In addition, a considerable literature suggests that psychological factors are important predictors of disability in chronic musculoskeletal pain (Boersma et al., 2014), whereas comparable research on other pain etiologies is less consistent (Molton et al., 2009; Osborne et al., 2007). Finally, gender and age differences in pain-related disability are regularly observed (Réthelyi et al., 2001; Stubbs et al., 2010). Despite evidence of racial/ethnic differences in pain-related disability (Chibnall & Tait, 2005; Murtaugh et al., 2017), race and ethnicity were not examined as potential moderators due to inconsistent and limited reporting across studies.

### Study Selection

Studies were included if they met the following criteria: (1) published in English in a peer-reviewed journal; (2) included participants who self-reported chronic pain (i.e., pain that persists or recurs for more than three months); (3) included self-report measures of chronic pain acceptance and pain-related disability; and (4) reported bivariate correlations (*r*) between chronic pain acceptance and pain-related disability (including sample size or degrees of freedom). No age limitations were applied as part of the inclusion criteria for this meta-analysis. Studies investigating treatment efficacy were only included if correlations were reported at baseline (i.e., prior to treatment). When studies described results for two or more distinct samples, results were coded separately for each. To maximize construct validity, studies that utilized measures of pain interference in lieu of pain-related disability were excluded. Despite sharing conceptual overlap, pain interference, as measured by the Brief Pain Inventory (Cleeland & Ryan, 1994) or West Haven-Yale Multidimensional Pain Inventory (Kerns et al., 1985), contains items on the influence of pain on mood and enjoyment of life and represents a broader construct (Guthrie et al., 2022). Indeed, research suggests pain interference and physical functioning are related but distinct constructs, demonstrating only weak associations across time (Karayannis et al., 2017).

### Study Quality Assessment

The methodological quality of included studies was assessed using a modified version of the Downs and Black checklist (Downs & Black, 1998). Consistent with previous research (Zadro et al., 2019), the modified checklist used here contained eight items relevant to the data under study. Items were rated as either “yes” (one point) or “no/unable to determine” (zero points), yielding a maximum possible score of 8. Higher scores indicated greater methodological quality.

### Screening and Data Extraction

One reviewer (KMW) performed title and abstract screening on all records. A second reviewer (ECL) independently screened a random 10% sample to assess the reliability of the screening (94% agreement). In the event of disagreement, articles were included for full-text review. KMW performed full-text review of all identified articles and ECL independently reviewed a random 10% sample to check reliability (100% agreement). Data were then independently extracted by each reviewer. For each study, the following information was recorded: (1) sample size; (2) correlation (*r*) between chronic pain acceptance and pain-related disability; (3) specific measures used; (4) pain type, duration, intensity, and treatment status; and (5) gender and age composition. For instances in which multiple chronic pain acceptance or pain-related disability measures were used, intercorrelations were also recorded. Pain type was coded as “musculoskeletal” or “other” (e.g., mixed etiology, sickle cell disease). Pain duration was coded as a continuous variable based on the average length of time participants had experienced pain, in months. Pain intensity was coded as the average pain rating on numerical rating or visual analog scales. Linear transformations were performed to convert all pain ratings to a single metric (i.e., 0–100). Treatment status was coded as a dichotomous variable (yes/no), where samples were considered treatment-seeking if recruited from a pain treatment program or seeking specialty treatment services at the time of study enrollment. Finally, gender composition was coded as the percentage of the sample that was female, and age was coded using the average reported age.

### Analytic Strategy

Analyses were conducted with Comprehensive Meta-Analysis, version 4 (Biostat, Englewood, NJ). Given the expected heterogeneity among included studies, a random effects model was employed. Correlational effect sizes (*r*) were converted to Fisher’s *z* scale and pooled using inverse variance weighting (Borenstein et al., 2021). Summary effects and their corresponding confidence intervals were converted back to correlations for presentation. To test whether the correlation varied across facets of chronic pain acceptance, analyses were repeated using individual CPAQ subscales as predictor measures. Non-overlapping 95% confidence intervals were considered significantly different.

To meet the assumption of independence, only one correlation between chronic pain acceptance and pain-related disability was included from each sample. For instances in which multiple chronic pain acceptance or pain-related disability measures were employed, composite formulas were utilized to create a single effect size (Nunnally, 1978). In the case of one study that lacked the necessary information to use composite formulas (Bendayan et al., 2012), the mean correlation between chronic pain acceptance and pain-related disability was calculated.

For analysis of categorical moderators, meta-analytic calculations were performed separately for each level of the variable. Consistent with the analytic strategy reported above, 95% confidence intervals were calculated for subgroups and compared to test for moderation. For continuous moderators, weighted least squares regression with method-of-moments parameters was employed (Lipsey & Wilson, 2001).

To assess availability bias, a file drawer analysis was conducted (Orwin, 1983). This analysis estimated the number of studies with null findings that would be required to reduce the mean correlation between chronic pain acceptance and pain-related disability to practical insignificance. A critical level of *r* = −.1 was used (Cohen, 1988).

## Results

### Study Selection

**Fig. 1:**
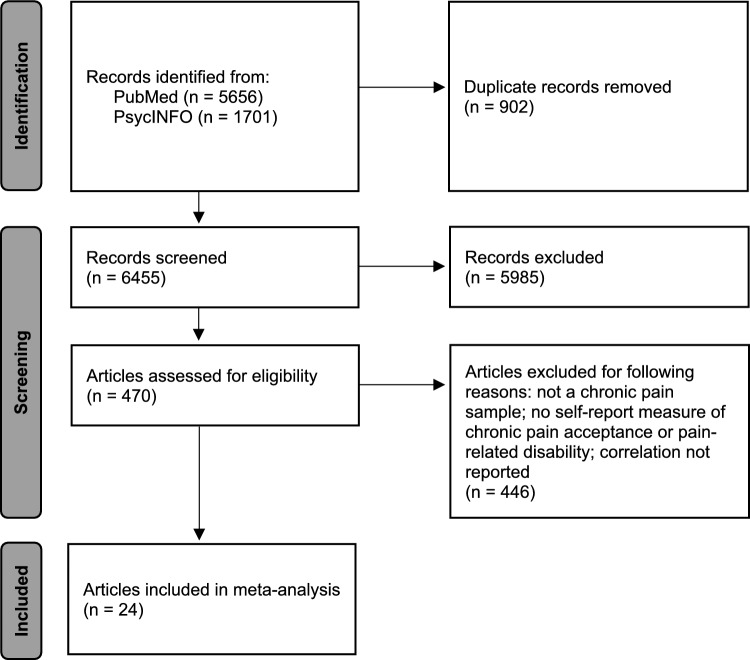
PRISMA flow diagram for study selection process

A PRISMA flow diagram for the study selection process is depicted in Fig. 1. Of the 7357 records identified as potentially relevant via database searches, 902 were found to be duplicates. Title/abstract screening was performed on the remaining 6455 records. Of these, 470 were deemed possibly eligible and received full-text review. Through this process, 446 articles were excluded for not meeting inclusion criteria. Thus, the current meta-analysis included 24 primary studies with 26 independent samples (*N* = 6072). The manual review of reference lists yielded no additional studies for inclusion.

### Study Characteristics

Table 1 displays the coded variables from each sample. Initial agreement between the two independent raters was 96%, and 100% consensus was achieved through discussion and reference to the original articles. Eligible studies were published between 2006 and 2020. Sample sizes ranged from 21 to 686, totaling 6072 individuals with chronic pain. The studies included adolescent and adult samples, with a mean age range from 14.5 to 54.6 years. Samples were drawn from a variety of countries, including the United States, Australia, Belgium, Brazil, Canada, England, Germany, Iran, Italy, Scotland, and Spain.

**Table 1:** Summary of coded information

Reference	*N*	Measures		*r*	Pain Characteristics				Demographics	
		Acceptance	Disability		Type	Duration (months)	Intensity (0–100)	Treatment Seeking	% F	Age
Baranoff et al., 2014	334	CPAQ-20; CPAQ-8	RMDQ	−.42	Other	97.50	66.80	Yes	57.40	46.19
Bendayan et al., 2012	86	CPAQ-20	IFI-Impair	−.44	Other	–	–	Yes	–	–
Connolly et al., 2019	128	CPAQ-A	FDI	−.62	Other	–	54.14	No	67.97	14.46
De Vlieger, et al., 2006	476	CPAQ-20; PaSol-Accept	PDI	−.39	Other	184.93	–	No	71.80	52.83
Gillanders et al., 2013	150	CPAQ-20	RMDQ	−.62	Other	120.00	–	No	66.00	50.80
Howard et al., 2017	303	CPAQ-20	ODI	−.37	MSK	–	61.94	Yes	39.93	46.42
Kanzler et al., 2019	207	CPAQ-20	ODI	−.63	Other	–	57.83	Yes	41.55	–
Matthie et al., 2020	170	CPAQ-8	CPGS-Dis	−.06	Other	–	62.94	No	53.53	28.05
McGarrigle et al., 2020	129	CPAQ-A	FDI	−.61	Other	–	53.76	No	68.22	14.45
Mesgarian et al., 2013	245	CPAQ-20	RMDQ	−.38	Other	69.60	68.33	Yes	72.80	43.80
Monticone et al., 2013	142	CPAQ-20	RMDQ	−.59	MSK	24.00	–	Yes	59.86	54.57
Nicholas & Asghari, 2006	252	CPAQ-20	RMDQ	−.38	Other	93.70	66.67	Yes	62.30	50.33
Ramírez-Maestre et al., 2012	299	CPAQ-20	IFI-Impair	−.40	MSK	25.21	52.50	No	53.85	44.18
Ramírez-Maestre & Esteve, 2014										
Sample 1	190	CPAQ-20	IFI-Impair	−.46	MSK	70.58	49.00	No	0.00	46.28
Sample 2	210	CPAQ-20	IFI-Impair	−.44	MSK	63.26	55.70	No	100.00	46.17
Ramírez-Maestre et al., 2014	686	CPAQ-20	IFI-Impair; RMDQ	−.29	MSK	48.70	52.25	No	59.04	45.40
Ruskin et al., 2017	21	CPAQ-20	FDI	−.25	Other	41.76	55.00	Yes	95.24	15.52
Sardá et al., 2009										
Sample 1	311	CPAQ-20	RMDQ	−.40	Other	87.00	62.00	Yes	73.95	48.90
Sample 2	311	CPAQ-20	RMDQ	−.23	Other	72.00	58.00	Yes	73.31	49.20
Serbic & Pincus, 2017	287	CPAQ-8	RMDQ	−.65	MSK	–	62.60	No	66.20	49.88
Sielski, et al., 2017	165	PaSol-Accept	PDI	−.11	MSK	159.60	58.00	Yes	60.00	53.00
Sutherland & Morley, 2008	82	CPAQ-20	PDI	−.45	Other	128.40	56.70	Yes	62.20	45.45
Timmers et al., 2019	578	CPAQ-A	FDI	−.57	Other	27.70	60.80	Yes	–	15.20
Wallace et al., 2011	109	CPAQ-A	FDI	−.63	Other	29.00	68.00	Yes	85.00	15.20
Weiss et al., 2013	112	CPAQ-A	FDI	−.50	Other	37.00	54.60	Yes	75.89	15.47
Wright et al., 2011	89	CPAQ-20	PDI	−.49	MSK	141.60	66.19	Yes	71.91	53.65

*CPAQ* Chronic Pain Acceptance Questionnaire, *PaSol-Accept* Pain Solutions Questionnaire, Acceptance of the Insolubility of Pain subscale, *CPGS-Dis* Chronic Pain Grade Scale, Disability subscale, *FDI* Functional Disability Inventory, *IFI-Impair* Impairment and Functioning Inventory, Impairment subscale, *ODI* Oswestry Disability Index, *PDI* Pain Disability Index, *RMDQ* Roland Morris Disability Questionnaire, *MSK* musculoskeletal, *% F* percent of sample that was female

### Correlation Between Chronic Pain Acceptance and Pain-Related Disability

**Fig. 2:**
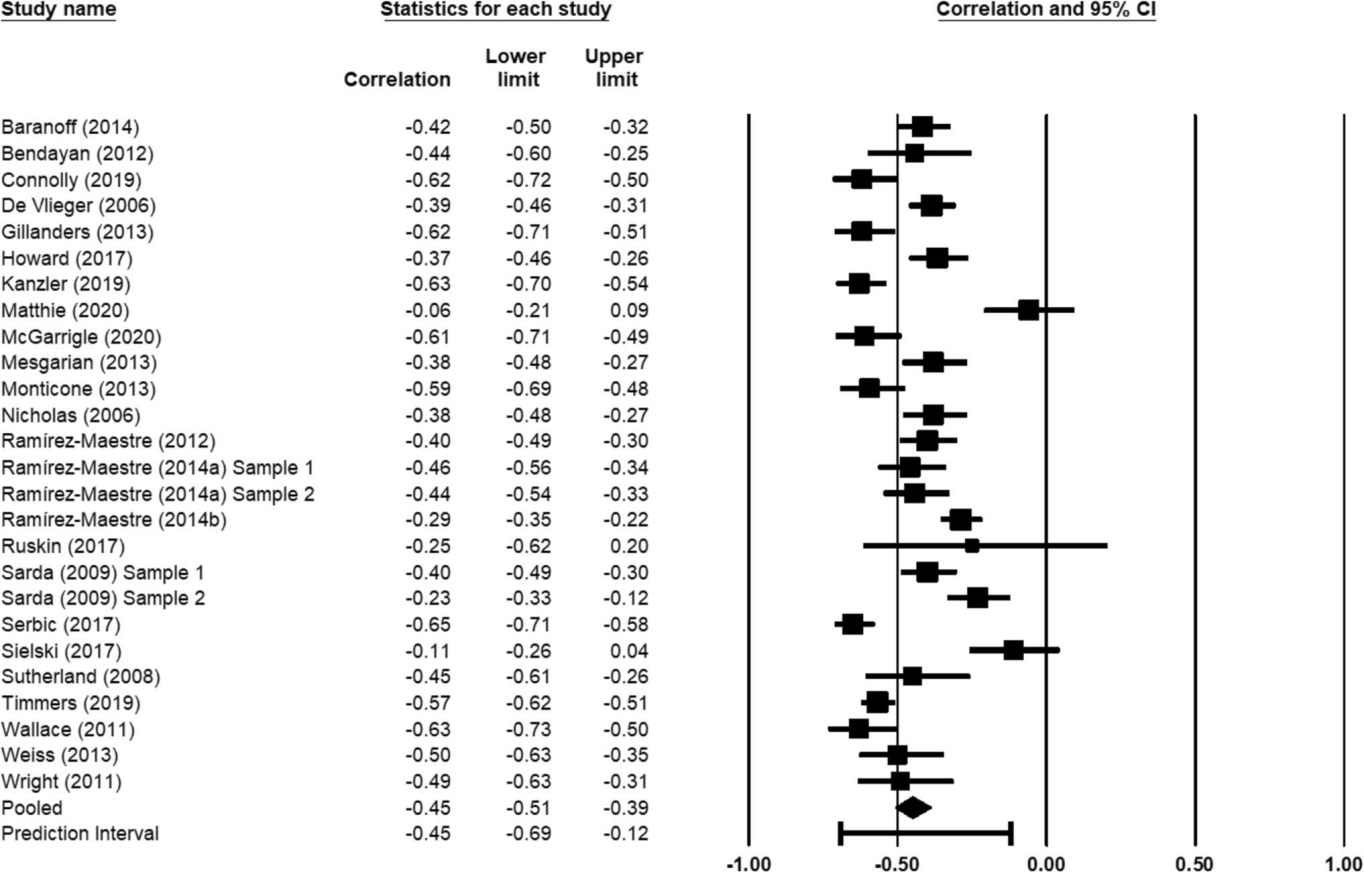
Forest plot for meta-analysis of the association between chronic pain acceptance and pain-related disability using random effects model

Results indicated that the weighted mean correlation between chronic pain acceptance and pain-related disability was −0.45 (95% CI −0.51, −0.39), an association that can be characterized as moderate in magnitude (Cohen, 1988). A forest plot of the distribution of effect sizes (*k* = 26) is presented in Fig. 2. Approximately 87% of the variance in observed effects reflected variance in true effects rather than sampling error. The 95% prediction interval, addressing between-study dispersion in effects sizes, was −0.69 to −0.12.

Correlations between the subscales of the CPAQ and pain-related disability were reported for 11 samples. When analyzed separately, the mean correlations using the Activity Engagement and Pain Willingness subscales were −0.44 (95% CI −0.51, −0.36) and −0.35 (95% CI −0.42, −0.27), respectively. Overlapping 95% confidence intervals suggested this was a non-significant difference. Full meta-analytic results are displayed in Table 2.

**Table 2:** Weighted mean correlations between chronic pain acceptance and pain-related disability, including subscale and categorical moderator analyses

Predictor	*N*	*k*	Random effects models				
			*r*	95% CI	*Q*	*I*^2^	τ^2^
Acceptance	6072	26	−.45	−.51, −.39	194.92	87.17	0.03
Activity Engagement	2833	11	−.44	−.51, −.36	55.36	81.94	0.02
Pain Willingness	2833	11	−.35	−.42, −.27	48.67	79.46	0.02
Moderator Analyses							
Acceptance Measure							
CPAQ-20	4394	18	−.43	−.48, −.38	71.43	76.20	0.01
CPAQ-8	791	3	−.41	−.67, −.06	54.69	96.34	0.11
CPAQ-A	1056	5	−.58	−.62, −.54	3.00	0.00	0.00
PaSol-Accept	641	2	−.12	−.19, −.04	0.01	0.00	0.00
Disability Measure							
FDI	1077	6	−.58	−.62, −.53	5.93	15.61	< 0.01
IFI-Impair	1471	5	−.40	−.46, −.32	8.77	54.41	0.01
ODI	510	2	−.51	−.72, −.21	15.39	93.50	0.06
PDI	812	4	−.36	−.50, −.20	14.97	79.97	0.03
RMDQ	2718	9	−.44	−.55, −.32	107.12	92.53	0.04
Pain Type							
Musculoskeletal	2371	9	−.43	−.53, −.32	74.56	89.27	0.03
Other	3701	17	−.46	−.53, −.39	116.03	86.21	0.03
Seeking Pain Treatment							
No	2725	10	−.47	−.56, −.36	102.44	91.22	0.04
Yes	3347	16	−.44	−.51, −.37	91.98	83.69	0.03

*k* number of effect sizes, *r* weighted mean correlation, *CI* confidence interval, *Q* Hedges’ Q test for homogeneity, *I*^2^ percentage of variation in observed effects due to heterogeneity rather than sampling error, *τ*^2^ between-study variance, *CPAQ* Chronic Pain Acceptance Questionnaire, *PaSol-Accept* Pain Solutions Questionnaire, Acceptance of the Insolubility of Pain subscale, *FDI* Functional Disability Inventory, *IFI-Impair* Impairment and Functioning Inventory, Impairment subscale, *ODI* Oswestry Disability Index, *PDI* Pain Disability Index, *RMDQ* Roland Morris Disability Questionnaire

### Moderator Analyses

#### Specific Measures

Mean correlations were stronger when measuring chronic pain acceptance with different versions of the CPAQ (*r* ranged from −.41 to −.58) compared to the Pain Solutions Questionnaire (PaSol; De Vlieger et al., 2006) Acceptance of the Insolubility of Pain subscale (*r* = −.12; see Table 2). On the basis of confidence intervals, these differences were significant for the CPAQ-20 and CPAQ-A (McCracken et al., 2004, 2010), but not the CPAQ-8 (Fish et al., 2010). Additionally, the confidence intervals for the CPAQ-20 and CPAQ-A were non-overlapping with each other. The CPAQ-A demonstrated a significantly stronger association with pain-related disability (*r* = −.58) than the CPAQ-20 (*r* = −.43). Together, these results suggest the acceptance-based instrument type moderated the association between chronic pain acceptance and pain-related disability.

With regard to the pain-related disability measures, only one study used the Chronic Pain Grade Questionnaire (CPGQ; Von Korff et al., 1992) Disability subscale, and was, therefore, excluded from the instrument-specific moderation analyses. The mean correlation between chronic pain acceptance and pain-related disability was significantly stronger when using the Functional Disability Inventory (FDI; Walker & Greene, 1991; *r* = −.58) compared to the Impairment and Functioning Inventory (IFI; Maestre & Velasco, 2003) Impairment subscale (*r* = −.40) and Pain Disability Index (PDI; Pollard, 1984; *r* = −.36). These findings suggest the type of pain-related disability measure also moderated the association between chronic pain acceptance and pain-related disability. Overlapping confidence intervals were observed for the remaining measures.

#### Pain Characteristics

Results indicated that the mean correlation between chronic pain acceptance and pain-related disability did not differ as a function of pain type or treatment status (see Table 2). Likewise, for the continuous pain characteristics, findings suggested that neither pain duration (*p* = .15) nor pain intensity (*p* = .99) moderated the association between chronic pain acceptance and pain-related disability.

#### Demographic Factors

Gender (% female) and age composition were each tested as continuous moderators. Results indicated that neither gender (*p* = .82) nor age (*p* = .12) moderated the association between chronic pain acceptance and pain-related disability.

#### Study Quality

Across studies, quality scores ranged from 5–8 out of 8 (*M* = 6.58, *SD* = 0.78). The validity category least often addressed was external validity. For example, only 10 studies provided specifics on how individuals were recruited and whether those asked to participate were representative of the source population from which they were drawn. Quality scores were found to be unrelated to the mean correlation between chronic pain acceptance and pain-related disability (*p* = .31). Full data on the study quality assessment sare presented in Supplementary Information.

### Availability Bias

File drawer analysis indicated that 95 studies with effect sizes of zero would be required to reduce the mean correlation between chronic pain acceptance and pain-related disability to practical insignificance. Therefore, in the context of the current meta-analysis comprising 26 samples, results were deemed unlikely to have emerged from a biased sampling of studies.

## Discussion

Research has demonstrated associations between chronic pain acceptance and pain-related disability (Kanzler et al., 2019; Nicholas & Asghari, 2006; Sardá et al., 2009). However, inconsistencies in the literature have prevented inferences regarding the strength of this relationship. Synthesizing findings from 6072 individuals with chronic pain across 26 samples, the current meta-analysis revealed a negative association between chronic pain acceptance and pain-related disability that can be characterized as moderate in magnitude (*r* = −.45; Cohen, 1988). Offering support for the robustness of the summary effect size, file drawer analysis suggested an additional 95 studies with null findings would be needed to reduce the correlation between chronic pain acceptance and pain-related disability to practical insignificance. These results are consistent with mounting evidence indicating that acceptance is a crucial aspect of functioning for persons living with chronic pain (McCracken & Vowles, 2006; Thompson & McCracken, 2011).

With regard to individual facets of chronic pain acceptance, the Activity Engagement subscale of the CPAQ was slightly more strongly associated with pain-related disability (*r* = −.44) than the Pain Willingness subscale (*r* = −.35). This was perhaps unsurprising given the apparent conceptual overlap between pain-related disability and items that constitute Activity Engagement. Indeed, researchers have recognized the possibility of convergence between measures of acceptance and disability (Lauwerier et al., 2015). However, it is important to note that the Activity Engagement subscale of the CPAQ does not assess overt behavior. Derived from a functional contextual perspective, the CPAQ measures the extent to which an individual’s behavior, in the presence of pain, is free from the cognitive-affective sequelae that can restrict engagement in life activities (McCracken et al., 2007). Further, 95% confidence intervals for correlations with pain-related disability overlapped for Activity Engagement and Pain Willingness, indicating a statistically non-significant difference between the facets of chronic pain acceptance.

The association between chronic pain acceptance and pain-related disability was stable across most of the moderators tested, including pain characteristics, demographic factors, and study quality. Concerning the specific acceptance measures used, there was evidence to suggest that instrument type moderated the strength of the relationship between chronic pain acceptance and pain-related disability. Specifically, the association was significantly stronger when using different versions of the CPAQ (i.e., CPAQ-20 and CPAQ-A) compared to the PaSol. Although the development of the PaSol was informed by the CPAQ (De Vlieger et al., 2006), it has been posited that different aspects of chronic pain acceptance may be captured by these measures (Lauwerier et al., 2015; Reneman et al., 2010), highlighting the nuanced and multifaceted nature of this construct and the challenges in its measurement. Relative to the different versions of the CPAQ, the PaSol Acceptance of the Insolubility of Pain subscale is also briefer, likely resulting in less comprehensive coverage of chronic pain acceptance. The correlation was also stronger when using the CPAQ-A compared to the CPAQ-20. While this difference may have been attributable to measure content, it is important to acknowledge that the CPAQ-A was only administered to adolescents. Thus, the stronger correlation observed for the CPAQ-A (vs. CPAQ-20) may have been a function of the age discrepancy between the groups of samples. Similarly, in the moderation analyses that grouped samples based on the pain-related disability measures employed, the association was strongest for the FDI, an instrument that was also used exclusively among adolescents. Nevertheless, age, when tested continuously, was not a significant moderator. Relationships between age and the experience of pain and disability are complex and vary across contexts (Gagliese & Melzack, 2003; Gibson & Helme, 2001), and additional research will be necessary to clarify the role of age in the association between chronic pain acceptance and pain-related disability.

The inclination to avoid pain is normative (Chapman et al., 2008), and learning to accept chronic pain may require assistance. An implication of this meta-analysis is that pain acceptance is likely a potent intervention target for pain-related disability in chronic pain patients. The current results are consistent with literature showing that increases in chronic pain acceptance are associated with improvements in functional outcomes following acceptance-based behavioral treatments (Huggins et al., 2012; McCracken et al., 2005; Simister et al., 2018). Interestingly, increases in chronic pain acceptance are even associated with reduction in pain intensity in some research, albeit to a lesser extent than reductions in disability (McCracken et al., 2005). Other implications of this meta-analysis center on the brevity and ease of administration of the chronic pain acceptance measures studied. Clinicians may consider using instruments like the CPAQ as a screening tool to detect patients at risk for developing pain-related disability following acute injury or during the initial stages of pain chronification. Indeed, chronic pain acceptance, assessed via the CPAQ, has been prospectively associated with disability up to one year post-assessment (Cook et al., 2015).

To our knowledge, this is the first meta-analytic synthesis of associations between chronic pain acceptance and pain-related disability. Other notable strengths include the large sample size and broad geographic representation across the primary studies, which enhance generalizability. However, several limitations should also be noted. First, pain-related disability was narrowly assessed in this study, strictly using self-report measures. Given that self-reported and performance-based assessments (e.g., timed sit-to-stand tests) may be differentially impacted by patient characteristics, future research will be necessary to clarify whether the strength of the association between chronic pain acceptance and pain-related disability varies as a function of assessment type. For example, psychological distress appears to make a unique contribution to self-reported disability, whereas performance-based disability is more closely linked to pain intensity, physical well-being, and symptom distribution (Wand et al., 2010). Second, acceptance is but one of the six core processes that constitute psychological flexibility in ACT (Hayes et al., 2006). Because these processes are believed to be interdependent and complimentary (Hayes et al., 2006), their combined influence will likely best assist patients in navigating the challenges imposed by chronic pain. Future work may benefit from examining the relationship between pain-related disability and psychological flexibility more broadly. Third, several subgroup analyses reported in this study were conducted using a small number of samples and need to be interpreted with caution. For example, two studies were used to calculate the correlation between chronic pain acceptance as measured by the PaSol and pain-related disability. Although this association appeared weaker than those observed using the CPAQ-20 and CPAQ-A, there is insufficient data to draw firm conclusions (Cuijpers et al., 2021). Fourth, it is important to acknowledge that the strength of the association between chronic pain acceptance and pain-related disability may be influenced by additional unmeasured variables not accounted for in primary studies. For example, in addition to conferring analgesia, the use of opioid medications can produce cognitive and affective side effects that may inhibit one’s ability to participate in the active and aware embrace of thoughts and feelings (Hayes et al., 2006; van Steenbergen et al., 2019), potentially weakening the relationship between chronic pain acceptance and pain-related disability. Similarly, psychological factors such as depression or anxiety might also moderate this association (Lerman et al., 2015). Fifth, only cross-sectional data were analyzed in this study, thus, precluding any causal interpretation of the association between chronic pain acceptance and pain-related disability. Although previous meta-analyses have highlighted the efficacy of ACT and related therapies on chronic pain outcomes such as disability (Ma et al., 2023; Veehof et al., 2016), future longitudinal research will be needed to clarify the temporal precedence of chronic pain acceptance and pain-related disability to better understand their interplay in the context of acceptance-based pain treatments. Sixth, pain types in this analysis were categorized as “musculoskeletal” or “other,” limiting the ability to detect varied association strengths between chronic pain acceptance and pain-related disability across a wider range of etiologies. Seventh, our literature search was limited to English-language publications in only two major databases (PubMed and PsycINFO), which may not encompass all relevant evidence. Finally, the lack of preregistration and the exclusion of unpublished data are notable limitations of this meta-analysis.

## Conclusion

Chronic pain remains a leading cause of physical disability worldwide, and an evolving literature suggests that ACT and other third-wave behavioral therapies may help ameliorate this public health concern. Consistent with the theoretical basis of these approaches, chronic pain acceptance has emerged as a vital aspect of functioning in persons living with chronic pain. The current meta-analysis extends previous research by providing the first synthesis of associations between chronic pain acceptance and pain-related disability. Overall, the results indicated a robust negative relationship that can be characterized as moderate in magnitude. Methodological limitations notwithstanding, the association between chronic pain acceptance and pain-related disability was found to be moderated by the measures used to assess these constructs. Identifying pain-related variables that impact the trajectory or outcomes of interventions targeting chronic pain acceptance should be a priority for future research.

## Supplementary Information

Below is the link to the electronic supplementary material.

Supplementary file1 (DOCX 13 KB)

Supplementary file2 (DOCX 14 KB)

Supplementary file3 (DOCX 17 KB)

## Data Availability

Institutional review board approval was not required for this work because it used previously published data.

## References

[CR1] Bahat, H. S., Weiss, P. L. T., Sprecher, E., Krasovsky, A., & Laufer, Y. (2014). Do neck kinematics correlate with pain intensity, neck disability or with fear of motion? *Manual Therapy,**19*, 252–258. 24291364 10.1016/j.math.2013.10.006

[CR2] Baranoff, J., Hanrahan, S. J., Kapur, D., & Connor, J. P. (2014). Validation of the chronic pain acceptance questionnaire-8 in an Australian pain clinic sample. *International Journal of Behavioral Medicine,**21*, 177–185. 23179676 10.1007/s12529-012-9278-6

[CR3] Bean, D. J., Johnson, M. H., & Kydd, R. R. (2014). Relationships between psychological factors, pain, and disability in complex regional pain syndrome and low back pain. *The Clinical Journal of Pain,**30*, 647–653. 24135903 10.1097/AJP.0000000000000007

[CR4] Bendayan, R., Esteve, R., & Blanca, M. J. (2012). New empirical evidence of the validity of the chronic pain acceptance questionnaire: The differential influence of activity engagement and pain willingness on adjustment to chronic pain. *British Journal of Health Psychology,**17*, 314–326. 22107355 10.1111/j.2044-8287.2011.02039.x

[CR5] Boersma, K., Carstens-Söderstrand, J., & Linton, S. J. (2014). From acute pain to chronic disability: Psychosocial processes in the development of chronic musculoskeletal pain and disability. *Handbook of musculoskeletal pain and disability disorders in the workplace* (pp. 205–217). Springer New York.

[CR6] Borenstein, M., Hedges, L. V., Higgins, J. P., & Rothstein, H. R. (2021). *Introduction to meta-analysis*. John Wiley & Sons.

[CR7] Brodke, D. S., Goz, V., Lawrence, B. D., Spiker, W. R., Neese, A., & Hung, M. (2017). Oswestry disability index: A psychometric analysis with 1,610 patients. *The Spine Journal,**17*, 321–327. 27693732 10.1016/j.spinee.2016.09.020

[CR8] Chapman, C. R., Tuckett, R. P., & Song, C. W. (2008). Pain and stress in a systems perspective: Reciprocal neural, endocrine, and immune interactions. *The Journal of Pain,**9*, 122–145. 18088561 10.1016/j.jpain.2007.09.006 PMC2278005

[CR9] Chibnall, J. T., & Tait, R. C. (2005). Disparities in occupational low back injuries: Predicting pain-related disability from satisfaction with case management in African Americans and Caucasians. *Pain Medicine,**6*, 39–48. 15669949 10.1111/j.1526-4637.2005.05003.x

[CR10] Cleeland, C., & Ryan, K. (1994). Pain assessment: Global use of the Brief Pain Inventory. *Annals of the Academy of Medicine, Singapore,**23*, 129–138. 8080219

[CR11] Cohen, J. (1988). *Statistical power analysis for the behavioral sciences* (2nd ed.). Lawrence Erlbaum Associates, Inc.

[CR12] Connolly, S., Ferreira, N., McGarrigle, L., & DeAmicis, L. (2019). Further validation of the chronic pain acceptance questionnaire for adolescents in a broader paediatric context. *Journal of Contextual Behavioral Science,**12*, 314–321.

[CR13] Cook, A. J., Meyer, E. C., Evans, L. D., Vowles, K. E., Klocek, J. W., Kimbrel, N. A., Gulliver, S. B., & Morissette, S. B. (2015). Chronic pain acceptance incrementally predicts disability in polytrauma-exposed veterans at baseline and 1-year follow-up. *Behaviour Research and Therapy,**73*, 25–32. 26233854 10.1016/j.brat.2015.07.003 PMC5032639

[CR14] Cuijpers, P., Griffin, J. W., & Furukawa, T. A. (2021). The lack of statistical power of subgroup analyses in meta-analyses: A cautionary note. *Epidemiology and Psychiatric Sciences,**30*, e78. 34852862 10.1017/S2045796021000664 PMC8679832

[CR15] De Vlieger, P., Van den Bussche, E., Eccleston, C., & Crombez, G. (2006). Finding a solution to the problem of pain: Conceptual formulation and the development of the pain solutions questionnaire (PaSol). *Pain,**123*, 285–293. 16675113 10.1016/j.pain.2006.03.005

[CR16] Downs, S. H., & Black, N. (1998). The feasibility of creating a checklist for the assessment of the methodological quality both of randomised and non-randomised studies of health care interventions. *Journal of Epidemiology & Community Health,**52*, 377–384. 9764259 10.1136/jech.52.6.377 PMC1756728

[CR17] Duyur Çakıt, B., Genç, H., Altuntaş, V., & Erdem, H. R. (2009). Disability and related factors in patients with chronic cervical myofascial pain. *Clinical Rheumatology,**28*, 647–654. 19224128 10.1007/s10067-009-1116-0

[CR18] Dworkin, R. H., Turk, D. C., Farrar, J. T., Haythornthwaite, J. A., Jensen, M. P., Katz, N. P., Kerns, R. D., Stucki, G., Allen, R. R., Bellamy, N., Carr, D. B., Chandler, J., Cowan, P., Dionne, R., Galer, B. S., Hertz, S., Jadad, A. R., Kramer, L. D., Manning, D. C., … Witter, J. (2005). Core outcome measures for chronic pain clinical trials: IMMPACT recommendations. *Pain,**113*, 9–19. 10.1016/j.pain.2004.09.012 15621359 10.1016/j.pain.2004.09.012

[CR19] Feinstein, A. B., Forman, E. M., Masuda, A., Cohen, L. L., Herbert, J. D., Nandini Moorthy, L., & Goldsmith, D. P. (2011). Pain intensity, psychological inflexibility, and acceptance of pain as predictors of functioning in adolescents with juvenile idiopathic arthritis: A preliminary investigation. *Journal of Clinical Psychology in Medical Settings,**18*, 291–298. 21630002 10.1007/s10880-011-9243-6

[CR20] Fish, R. A., McGuire, B., Hogan, M., Morrison, T. G., & Stewart, I. (2010). Validation of the chronic pain acceptance questionnaire (CPAQ) in an Internet sample and development and preliminary validation of the CPAQ-8. *Pain,**149*, 435–443. 20188472 10.1016/j.pain.2009.12.016

[CR21] Fordyce, W. E. (1976). *Behavioral methods for chronic pain and Illness*. Mosby.

[CR22] Gagliese, L., & Melzack, R. (2003). Age-related differences in the qualities but not the intensity of chronic pain. *Pain,**104*, 597–608. 12927632 10.1016/S0304-3959(03)00117-9

[CR23] Garbi, Md. O. S. S., Hortense, P., Gomez, R. R. F., Silva, Td. CRd., Castanho, A. C. F., & Sousa, F. A. E. F. (2014). Pain intensity, disability and depression in individuals with chronic back pain. *Revista Latino-Americana De Enfermagem,**22*, 569–575. 25296139 10.1590/0104-1169.3492.2453 PMC4292655

[CR24] Gaskin, D. J., & Richard, P. (2012). The economic costs of pain in the United States. *The Journal of Pain,**13*, 715–724. 22607834 10.1016/j.jpain.2012.03.009

[CR25] Gibson, S. J., & Helme, R. D. (2001). Age-related differences in pain perception and report. *Clinics in Geriatric Medicine,**17*, 433–456. 11459714 10.1016/s0749-0690(05)70079-3

[CR26] Gillanders, D. T., Ferreira, N., Bose, S., & Esrich, T. (2013). The relationship between acceptance, catastrophizing and illness representations in chronic pain. *European Journal of Pain,**17*, 893–902. 23169693 10.1002/j.1532-2149.2012.00248.x

[CR27] Goldberg, D. S., & McGee, S. J. (2011). Pain as a global public health priority. *BMC Public Health,**11*, 770. 21978149 10.1186/1471-2458-11-770 PMC3201926

[CR28] Grönblad, M., Hupli, M., Wennerstrand, P., Järvinen, E., Lukinmaa, A., Kouri, J.-P., & Karaharju, E. O. (1993). Intercorrelation and test-retest reliability of the pain disability index (PDI) and the oswestry disability questionnaire (ODQ) and their correlation with pain intensity in low back pain patients. *The Clinical Journal of Pain,**9*, 189–195. 8219519 10.1097/00002508-199309000-00006

[CR29] Guthrie, D., Boring, B. L., Maffly-Kipp, J., Mathur, V. A., & Hicks, J. A. (2022). The experience of meaning in life in the context of pain-related disability. The positive psychology of personal factors: Implications for understanding disability, pp. 171–192.

[CR30] Hay, S. I., Abajobir, A. A., Abate, K. H., Abbafati, C., Abbas, K. M., Abd-Allah, F., Abdulkader, R. S., Abdulle, A. M., Abebo, T. A., & Abera, S. F. (2017). Global, regional, and national disability-adjusted life-years (DALYs) for 333 diseases and injuries and healthy life expectancy (HALE) for 195 countries and territories, 1990–2016: A systematic analysis for the global burden of disease study 2016. *The Lancet,**390*, 1260–1344. 10.1016/S0140-6736(17)32130-X PMC5605707 28919118

[CR31] Hayes, S. C., & Duckworth, M. P. (2006). Acceptance and commitment therapy and traditional cognitive behavior therapy approaches to pain. *Cognitive and Behavioral Practice*. 10.1016/j.cbpra.2006.04.002

[CR32] Hayes, S. C., Luoma, J. B., Bond, F. W., Masuda, A., & Lillis, J. (2006). Acceptance and commitment therapy: Model, processes and outcomes. *Behaviour Research and Therapy,**44*, 1–25. 16300724 10.1016/j.brat.2005.06.006

[CR33] Howard, K. J., Castaneda, R. A., Gray, A. L., Haskard-Zolnierek, K. B., & Jordan, K. (2017). Psychosocial factors related to functional restoration treatment completion and return-to-function for patients with chronic disabling occupational musculoskeletal disorders. *Journal of Occupational and Environmental Medicine,**59*, 320–326. 28267103 10.1097/JOM.0000000000000953

[CR34] Huggins, J. L., Bonn-Miller, M. O., Oser, M. L., Sorrell, J. T., & Trafton, J. A. (2012). Pain anxiety, acceptance, and outcomes among individuals with HIV and chronic pain: A preliminary investigation. *Behaviour Research and Therapy,**50*, 72–78. 22088609 10.1016/j.brat.2011.10.008

[CR35] Hughes, L. S., Clark, J., Colclough, J. A., Dale, E., & McMillan, D. (2017). Acceptance and commitment therapy (ACT) for chronic pain. *The Clinical Journal of Pain,**33*, 552–568. 27479642 10.1097/AJP.0000000000000425

[CR36] Jerome, A., & Gross, R. T. (1991). Pain disability index: construct and discriminant validity. *Archives of Physical Medicine and Rehabilitation,**72*, 920–922. 10.1016/0003-9993(91)90012-8 1929812 10.1016/0003-9993(91)90012-8

[CR37] Kanzler, K. E., Pugh, J. A., McGeary, D. D., Hale, W. J., Mathias, C. W., Kilpela, L. S., Karns-Wright, T. E., Robinson, P. J., Dixon, S. A., & Bryan, C. J. (2019). Mitigating the effect of pain severity on activity and disability in patients with chronic pain: The crucial context of acceptance. *Pain Medicine,**20*, 1509–1518. 30590737 10.1093/pm/pny197 PMC6686120

[CR38] Karayannis, N. V., Sturgeon, J. A., Chih-Kao, M., Cooley, C., & Mackey, S. C. (2017). Pain interference and physical function demonstrate poor longitudinal association in people living with pain: A PROMIS investigation. *Pain,**158*, 1063–1068. 28221284 10.1097/j.pain.0000000000000881 PMC5427986

[CR39] Kerns, R. D., Turk, D. C., & Rudy, T. E. (1985). The west haven-yale multidimensional pain inventory (WHYMPI). *Pain,**23*, 345–356. 4088697 10.1016/0304-3959(85)90004-1

[CR40] Kopec, J. A., Esdaile, J. M., Abrahamowicz, M., Abenhaim, L., Wood-Dauphinee, S., Lamping, D. L., & Williams, J. I. (1996). The Quebec back pain disability scale: Conceptualization and development. *Journal of Clinical Epidemiology,**49*, 151–161. 10.1016/0895-4356(96)00526-4 8606316 10.1016/0895-4356(96)00526-4

[CR41] Lauwerier, E., Caes, L., Van Damme, S., Goubert, L., Rosseel, Y., & Crombez, G. (2015). Acceptance: What’s in a name? A content analysis of acceptance instruments in individuals with chronic pain. *The Journal of Pain,**16*, 306–317. 25584430 10.1016/j.jpain.2015.01.001

[CR42] Lerman, S. F., Rudich, Z., Brill, S., Shalev, H., & Shahar, G. (2015). Longitudinal associations between depression, anxiety, pain, and pain-related disability in chronic pain patients. *Psychosomatic Medicine,**77*, 333–341. 25849129 10.1097/PSY.0000000000000158

[CR43] Lipsey, M. W., & Wilson, D. (2001). *Practical meta-analysis*. Sage.

[CR44] Ma, T. W., Yuen, A. S. K., & Yang, Z. (2023). The efficacy of acceptance and commitment therapy for chronic pain: A systematic review and meta-analysis. *The Clinical Journal of Pain,**39*, 147–157. 36827194 10.1097/AJP.0000000000001096

[CR45] Maestre, C. R., & Velasco, Y. V. (2003). Evaluación del funcionamiento diario en pacientes con dolor crónico. Psicología Conductual Revista Internacional de Psicología Clínica de la Salud.

[CR46] Matthie, N., Jenerette, C., Gibson, A., Paul, S., Higgins, M., & Krishnamurti, L. (2020). Prevalence and predictors of chronic pain intensity and disability among adults with sickle cell disease. *Health Psychology Open,**7*, 2055102920917250. 32426150 10.1177/2055102920917250 PMC7218472

[CR47] McCracken, L. M., Gauntlett-Gilbert, J., & Eccleston, C. (2010). Acceptance of pain in adolescents with chronic pain: Validation of an adapted assessment instrument and preliminary correlation analyses. *European Journal of Pain,**14*, 316–320. 19477144 10.1016/j.ejpain.2009.05.002

[CR48] McCracken, L. M., & Vowles, K. E. (2006). Acceptance of chronic pain. *Current Pain and Headache Reports,**10*, 90–94. 16539860 10.1007/s11916-006-0018-y

[CR49] McCracken, L. M., Vowles, K. E., & Eccleston, C. (2004). Acceptance of chronic pain: Component analysis and a revised assessment method. *Pain,**107*, 159–166. 14715402 10.1016/j.pain.2003.10.012

[CR50] McCracken, L. M., Vowles, K. E., & Eccleston, C. (2005). Acceptance-based treatment for persons with complex, long standing chronic pain: A preliminary analysis of treatment outcome in comparison to a waiting phase. *Behaviour Research and Therapy,**43*, 1335–1346. 16086984 10.1016/j.brat.2004.10.003

[CR51] McCracken, L., Vowles, K., & Thompson, M. (2007). Comment on Nicholas and Asghari: Pain 2006; 124: 269–79. *Pain,**128*, 283–284. 17123733 10.1016/j.pain.2006.10.002

[CR52] McGarrigle, L., Wesson, C., DeAmicis, L., Connoly, S., & Ferreira, N. (2020). Psychological mediators in the relationship between paediatric chronic pain and adjustment: An investigation of acceptance, catastrophising and kinesiophobia. *Journal of Contextual Behavioral Science,**18*, 294–305.

[CR53] Mesgarian, F., Asghari, A., Shaeiri, M. R., & Broumand, A. (2013). The persian version of the chronic pain acceptance questionnaire. *Clinical Psychology & Psychotherapy,**20*, 350–358. 22281840 10.1002/cpp.1769

[CR54] Molton, I. R., Stoelb, B. L., Jensen, M. P., Ehde, D. M., Raichle, K. A., & Cardenas, D. D. (2009). Psychosocial factors and adjustment to chronic pain in spinal cord injury: Replication and cross-validation. *Journal of Rehabilitation Research and Development,**46*, 31. 19533518 10.1682/jrrd.2008.03.0044 PMC2743728

[CR55] Monticone, M., Ferrante, S., Rocca, B., Nava, T., Parini, C., & Cerri, C. (2013). Chronic pain acceptance questionnaire: Confirmatory factor analysis, reliability, and validity in Italian subjects with chronic low back pain. *Spine,**38*, E824–E831. 23524871 10.1097/BRS.0b013e3182917299

[CR56] Murtaugh, C. M., Beissner, K. L., Barrón, Y., Trachtenberg, M. A., Bach, E., Henderson, C. R., Jr., Sridharan, S., & Reid, M. C. (2017). Pain and function in home care: A need for treatment tailoring to reduce disparities? *The Clinical Journal of Pain,**33*, 300–309. 27518494 10.1097/AJP.0000000000000410 PMC5473030

[CR57] Nicholas, M. K., & Asghari, A. (2006). Investigating acceptance in adjustment to chronic pain: Is acceptance broader than we thought? *Pain,**124*, 269–279. 16934925 10.1016/j.pain.2006.04.032

[CR58] Nunnally, J. C. (1978). *Psychometric theory* (2nd ed.). New York: McGraw-Hill.

[CR59] Orwin, R. G. (1983). A fail-safe N for effect size in meta-analysis. *Journal of Educational Statistics,**8*, 157–159.

[CR60] Osborne, T. L., Jensen, M. P., Ehde, D. M., Hanley, M. A., & Kraft, G. (2007). Psychosocial factors associated with pain intensity, pain-related interference, and psychological functioning in persons with multiple sclerosis and pain. *Pain,**127*, 52–62. 10.1016/j.pain.2006.07.017 16950570 10.1016/j.pain.2006.07.017

[CR61] Page, M. J., McKenzie, J. E., Bossuyt, P. M., Boutron, I., Hoffmann, T. C., Mulrow, C. D., Shamseer, L., Tetzlaff, J. M., Akl, E. A., & Brennan, S. E. (2021). The PRISMA 2020 statement: An updated guideline for reporting systematic reviews. *Systematic Reviews,**10*, 1–11. 33781348 10.1186/s13643-021-01626-4 PMC8008539

[CR62] Phillips, C. J. (2009). The cost and burden of chronic pain. *Reviews in Pain,**3*, 2–5. 26526940 10.1177/204946370900300102 PMC4590036

[CR63] Pollard, C. A. (1984). Preliminary validity study of the pain disability index. *Perceptual and Motor Skills,**59*, 974. 6240632 10.2466/pms.1984.59.3.974

[CR64] Ramírez-Maestre, C., & Esteve, R. (2014). The role of sex/gender in the experience of pain: resilience, fear, and acceptance as central variables in the adjustment of men and women with chronic pain. *The Journal of Pain,**15*, 608-618.e1. 24632112 10.1016/j.jpain.2014.02.006

[CR65] Ramírez-Maestre, C., Esteve, R., & López, A. E. (2012). The path to capacity: Resilience and spinal chronic pain. *Spine,**37*, E251–E258. 21857397 10.1097/BRS.0b013e31822e93ab

[CR66] Ramírez-Maestre, C., Esteve, R., & López-Martínez, A. (2014). Fear-avoidance, pain acceptance and adjustment to chronic pain: A cross-sectional study on a sample of 686 patients with chronic spinal pain. *Annals of Behavioral Medicine,**48*, 402–410. 24722965 10.1007/s12160-014-9619-6

[CR67] Reneman, M. F., Dijkstra, A., Geertzen, J. H., & Dijkstra, P. U. (2010). Psychometric properties of chronic pain acceptance questionnaires: A systematic review. *European Journal of Pain,**14*, 457–465. 19819172 10.1016/j.ejpain.2009.08.003

[CR68] Réthelyi, J. M., Berghammer, R., & Kopp, M. S. (2001). Comorbidity of pain-associated disability and depressive symptoms in connection with sociodemographic variables: Results from a cross-sectional epidemiological survey in Hungary. *Pain,**93*, 115–121. 11427322 10.1016/S0304-3959(01)00301-3

[CR69] Rice, A. S., Smith, B. H., & Blyth, F. M. (2016). Pain and the global burden of disease. *Pain,**157*, 791–796. 26670465 10.1097/j.pain.0000000000000454

[CR70] Roland, M., & Fairbank, J. (2000). The roland-morris disability questionnaire and the oswestry disability questionnaire. *Spine,**25*, 3115–3124. 10.1097/00007632-200012150-00006 11124727 10.1097/00007632-200012150-00006

[CR71] Ruskin, D. A., Gagnon, M. M., Kohut, S. A., Stinson, J. N., & Walker, K. S. (2017). A mindfulness program adapted for adolescents with chronic pain: Feasibility, acceptability, and initial outcomes. *The Clinical Journal of Pain,**33*, 1019–1029. 28328699 10.1097/AJP.0000000000000490

[CR72] Sardá, J., Jr., Nicholas, M. K., Asghari, A., & Pimenta, C. A. (2009). The contribution of self-efficacy and depression to disability and work status in chronic pain patients: A comparison between Australian and Brazilian samples. *European Journal of Pain,**13*, 189–195. 18448371 10.1016/j.ejpain.2008.03.008

[CR73] Serbic, D., & Pincus, T. (2017). The relationship between pain, disability, guilt and acceptance in low back pain: A mediation analysis. *Journal of Behavioral Medicine,**40*, 651–658. 28155002 10.1007/s10865-017-9826-2 PMC5501894

[CR74] Sielski, R., Glombiewski, J. A., Rief, W., Crombez, G., & Barke, A. (2017). Cross-cultural adaptation of the German Pain Solutions Questionnaire: an instrument to measure assimilative and accommodative coping in response to chronic pain. *Journal of Pain Research,**10*, 1437–1446. 28684921 10.2147/JPR.S130016 PMC5484560

[CR75] Simister, H. D., Tkachuk, G. A., Shay, B. L., Vincent, N., Pear, J. J., & Skrabek, R. Q. (2018). Randomized controlled trial of online acceptance and commitment therapy for fibromyalgia. *The Journal of Pain,**19*, 741–753. 29481976 10.1016/j.jpain.2018.02.004

[CR76] Stubbs, D., Krebs, E., Bair, M., Damush, T., Wu, J., Sutherland, J., & Kroenke, K. (2010). Sex differences in pain and pain-related disability among primary care patients with chronic musculoskeletal pain. *Pain Medicine,**11*, 232–239. 20002591 10.1111/j.1526-4637.2009.00760.x

[CR77] Sutherland, R., & Morley, S. (2008). Self-pain enmeshment: Future possible selves, sociotropy, autonomy and adjustment to chronic pain. *Pain,**137*, 366–377. 17977661 10.1016/j.pain.2007.09.023

[CR78] Thompson, M., & McCracken, L. M. (2011). Acceptance and related processes in adjustment to chronic pain. *Current Pain and Headache Reports,**15*, 144–151. 21222244 10.1007/s11916-010-0170-2

[CR79] Timmers, I., Simons, L. E., Hernandez, J. M., McCracken, L. M., & Wallace, D. P. (2019). Parent psychological flexibility in the context of pediatric pain: Brief assessment and associations with parent behaviour and child functioning. *European Journal of Pain,**23*, 1340–1350. 31002473 10.1002/ejp.1403

[CR80] Turk, D. C., Meichenbaum, D., & Genest, M. (1983). *Pain and behavioral medicine: A cognitive-behavioral perspective* (Vol. 1). New York: Guilford Press.

[CR81] Turner, J. A., & Romano, J. M. (2001). Cognitive-behavioral therapy for chronic pain. *Bonica’s management of pain* (3rd ed., pp. 1751–1758). Philadelphia: Lippincott Williams & Wilkins.

[CR82] van Steenbergen, H., Eikemo, M., & Leknes, S. (2019). The role of the opioid system in decision making and cognitive control: A review. *Cognitive, Affective, & Behavioral Neuroscience,**19*, 435–458. 10.3758/s13415-019-00710-6 PMC6599188 30963411

[CR83] Veehof, M. M., Trompetter, H. R., Bohlmeijer, E. T., & Schreurs, K. (2016). Acceptance-and mindfulness-based interventions for the treatment of chronic pain: A meta-analytic review. *Cognitive Behaviour Therapy,**45*, 5–31. 26818413 10.1080/16506073.2015.1098724

[CR84] Von Korff, M., Ormel, J., Keefe, F. J., & Dworkin, S. F. (1992). Grading the severity of chronic pain. *Pain,**50*, 133–149. 1408309 10.1016/0304-3959(92)90154-4

[CR85] Vos, T., Lim, S. S., Abbafati, C., Abbas, K. M., Abbasi, M., Abbasifard, M., Abbasi-Kangevari, M., Abbastabar, H., Abd-Allah, F., & Abdelalim, A. (2020). Global burden of 369 diseases and injuries in 204 countries and territories, 1990–2019: A systematic analysis for the Global Burden of Disease Study 2019. *The Lancet,**396*, 1204–1222. 10.1016/S0140-6736(20)30925-9 PMC7567026 33069326

[CR86] Vowles, K. E., McCracken, L. M., & Eccleston, C. (2007). Processes of change in treatment for chronic pain: The contributions of pain, acceptance, and catastrophizing. *European Journal of Pain,**11*, 779–787. 17303452 10.1016/j.ejpain.2006.12.007

[CR87] Walker, L. S., & Greene, J. W. (1991). The functional disability inventory: Measuring a neglected dimension of child health status. *Journal of Pediatric Psychology,**16*, 39–58. 1826329 10.1093/jpepsy/16.1.39

[CR88] Wallace, D. P., Harbeck-Weber, C., Whiteside, S. P., & Harrison, T. E. (2011). Adolescent acceptance of pain: Confirmatory factor analysis and further validation of the chronic pain acceptance questionnaire, adolescent version. *The Journal of Pain,**12*, 591–599. 21429810 10.1016/j.jpain.2010.11.004

[CR89] Wand, B. M., Chiffelle, L. A., O’Connell, N. E., McAuley, J. H., & DeSouza, L. H. (2010). Self-reported assessment of disability and performance-based assessment of disability are influenced by different patient characteristics in acute low back pain. *European Spine Journal,**19*, 633–640. 19851791 10.1007/s00586-009-1180-9 PMC2899836

[CR90] Weiss, K. E., Hahn, A., Wallace, D. P., Biggs, B., Bruce, B. K., & Harrison, T. E. (2013). Acceptance of pain: Associations with depression, catastrophizing, and functional disability among children and adolescents in an interdisciplinary chronic pain rehabilitation program. *Journal of Pediatric Psychology,**38*, 756–765. 23685451 10.1093/jpepsy/jst028

[CR91] Wright, M. A., Wren, A. A., Somers, T. J., Goetz, M. C., Fras, A. M., Huh, B. K., Rogers, L. L., & Keefe, F. J. (2011). Pain acceptance, hope, and optimism: Relationships to pain and adjustment in patients with chronic musculoskeletal pain. *The Journal of Pain,**12*, 1155–1162. 21820969 10.1016/j.jpain.2011.06.002

[CR92] Yong, R. J., Mullins, P. M., & Bhattacharyya, N. (2022). Prevalence of chronic pain among adults in the United States. *Pain,**163*, e328–e332. 33990113 10.1097/j.pain.0000000000002291

[CR93] Zadro, J., O’Keeffe, M., & Maher, C. (2019). Do physical therapists follow evidence-based guidelines when managing musculoskeletal conditions? Systematic review. *British Medical Journal Open,**9*, e032329. 10.1136/bmjopen-2019-032329 PMC6797428 31591090

