# Supplementary material for: The Association Between Chronic Pain Acceptance and Pain-Related Disability: A Meta-Analysis
Source: J Clin Psychol Med Settings. 2024 Dec 16;32(3):448–59. doi: 10.1007/s10880-024-10061-1 (PMC12370564; doi:10.1007/s10880-024-10061-1)
Supplement: Supplementary file 1 — Supplementary file1 (DOCX 13 KB) [file 10880_2024_10061_MOESM1_ESM.docx]

| **Supplementary Information.** Complete search strategy |
| --- |
|  |
| **PubMed**  ("pain"[MeSH Terms] OR "chronic pain"[MeSH Terms] OR ("pain"[Title/Abstract] OR "painful"[Title/Abstract])) AND ("adaptation, psychological"[MeSH Terms] OR "self-efficacy"[MeSH Terms] OR "health knowledge, attitudes, practice"[MeSH Terms] OR ("acceptance"[Title/Abstract] OR "accepting"[Title/Abstract] OR "accept"[Title/Abstract] OR "self-efficacy"[Title/Abstract])) AND ("disabled persons"[MeSH Terms] OR "disabled children"[MeSH Terms] OR "disability evaluation"[MeSH Terms] OR ("disability"[Title/Abstract] OR "function"[Title/Abstract] OR "functional"[Title/Abstract] OR "functioning"[Title/Abstract] OR "dysfunction"[Title/Abstract]))  **PsycINFO**  ( (DE "Pain") OR (DE "Chronic Pain") ) OR TI ( pain OR painful ) OR AB ( pain OR painful ) AND ( ((DE "Emotional Adjustment") OR (DE "Self-Efficacy")) OR (DE "Health Attitudes") ) OR TI ( acceptance OR accepting OR accept OR self-efficacy ) OR AB ( acceptance OR accepting OR accept OR self-efficacy ) AND ( ((DE "Functional Status") OR (DE "Activities of Daily Living")) OR (DE "Health Related Quality of Life") ) OR TI ( disability OR function OR functional OR functioning OR dysfunction ) OR AB ( disability OR function OR functional OR functioning OR dysfunction ) |
