# Supplementary material for: The Association Between Chronic Pain Acceptance and Pain-Related Disability: A Meta-Analysis
Source: J Clin Psychol Med Settings. 2024 Dec 16;32(3):448–59. doi: 10.1007/s10880-024-10061-1 (PMC12370564; doi:10.1007/s10880-024-10061-1)
Supplement: Supplementary file 2 — Supplementary file2 (DOCX 14 KB) [file 10880_2024_10061_MOESM2_ESM.docx]

Supplementary Information

Measures of Chronic Pain Acceptance and Pain-Related Disability

| Measure | | Description |
| --- | --- | --- |
| Chronic Pain Acceptance | | |
|  | CPAQ-20 | 20-item measure comprised of two subscales (Pain Willingness and Activity Engagement); assesses willingness to experience pain while continuing valued life activities; designed for adults |
|  | CPAQ-8 | 8-item measure comprised of two subscales (Pain Willingness and Activity Engagement); assesses willingness to experience pain while continuing valued life activities; short-form of CPAQ-20 |
|  | CPAQ-A | 20-item measure comprised of two subscales (Pain Willingness and Activity Engagement); assesses willingness to experience pain while continuing valued life activities; adapted for adolescents from CPAQ-20 |
|  | PaSol-Accept | The Acceptance of the Insolubility of Pain subscale assesses ability to disengage from problem-solving attempts and accept the uncontrollability of pain |
| Pain-Related Disability | | |
|  | CPGS-Dis | General measure; the Disability subscale assesses difficulty completing daily, recreational, social, and occupational activities |
|  | FDI | General measure; assesses difficulty completing daily, social, and physical activities |
|  | IFI-Impair | General measure; the Impairment subscale assesses difficulty completing household, autonomous behavior, leisure, and social activities |
|  | ODI | Specific to back pain, assesses difficulty completing self-care, physical, social, and occupational activities |
|  | PDI | General measure; assesses difficulty completing household, recreational, social, occupational, sexual, self-care, and life-support activities |
|  | RMDQ | Specific to back pain, assesses difficulty completing self-care, physical, and household activities |
| *Note.* CPAQ = Chronic Pain Acceptance Questionnaire; PaSol-Accept = Pain Solutions Questionnaire, Acceptance of the Insolubility of Pain subscale; CPGS-Dis = Chronic Pain Grade Scale, Disability subscale; FDI = Functional Disability Inventory; IFI-Impair = Impairment and Functioning Inventory, Impairment subscale; ODI = Oswestry Disability Index; PDI = Pain Disability Index; RMDQ = Roland Morris Disability Questionnaire | | |
