# Supplementary material for: The Association Between Chronic Pain Acceptance and Pain-Related Disability: A Meta-Analysis
Source: J Clin Psychol Med Settings. 2024 Dec 16;32(3):448–59. doi: 10.1007/s10880-024-10061-1 (PMC12370564; doi:10.1007/s10880-024-10061-1)
Supplement: Supplementary file 3 — Supplementary file3 (DOCX 17 KB) [file 10880_2024_10061_MOESM3_ESM.docx]

Supplementary Information

Study Quality Assessment

|  | Study Quality Items | | | | | | | | |
| --- | --- | --- | --- | --- | --- | --- | --- | --- | --- |
| Study | Item 1 | Item 2 | Item 3 | Item 4 | Item 5 | Item 6 | Item 7 | Item 8 | Total |
| Baranoff (2014) | 1 | 1 | 0 | 1 | 0 | 1 | 1 | 1 | 6 |
| Bendayan (2012) | 1 | 1 | 1 | 0 | 1 | 1 | 1 | 1 | 7 |
| Connolly (2019) | 1 | 1 | 1 | 1 | 0 | 0 | 1 | 1 | 6 |
| De Vlieger (2004) | 1 | 1 | 0 | 1 | 0 | 0 | 1 | 1 | 5 |
| Gillanders (2013) | 1 | 1 | 1 | 1 | 0 | 1 | 1 | 1 | 7 |
| Howard (2017) | 1 | 1 | 1 | 1 | 0 | 0 | 1 | 1 | 6 |
| Kanzler (2019) | 1 | 1 | 1 | 1 | 1 | 0 | 1 | 1 | 7 |
| Matthie (2020) | 1 | 1 | 1 | 1 | 1 | 0 | 1 | 1 | 7 |
| McGarrigle (2020) | 1 | 1 | 1 | 1 | 0 | 0 | 1 | 1 | 6 |
| Mesgarian (2013) | 1 | 1 | 1 | 1 | 0 | 0 | 1 | 1 | 6 |
| Monticone (2013) | 1 | 1 | 1 | 1 | 0 | 0 | 1 | 1 | 6 |
| Nicholas (2006) | 1 | 1 | 0 | 1 | 1 | 1 | 1 | 1 | 7 |
| Ramírez-Maestre (2012) | 1 | 1 | 1 | 1 | 0 | 0 | 1 | 1 | 6 |
| Ramírez-Maestre (2014a) | 1 | 1 | 1 | 1 | 1 | 1 | 1 | 1 | 8 |
| Ramírez-Maestre (2014b) | 1 | 1 | 1 | 0 | 1 | 0 | 1 | 1 | 6 |
| Ruskin (2017) | 1 | 1 | 1 | 1 | 1 | 1 | 0 | 1 | 7 |
| Sarda (2009) | 1 | 1 | 1 | 1 | 0 | 0 | 1 | 1 | 6 |
| Serbic (2017) | 1 | 1 | 1 | 1 | 0 | 0 | 1 | 1 | 6 |
| Sielski (2017) | 1 | 1 | 0 | 1 | 0 | 1 | 1 | 1 | 6 |
| Sutherland (2008) | 1 | 1 | 1 | 1 | 0 | 1 | 1 | 1 | 7 |
| Timmers (2019) | 1 | 1 | 1 | 1 | 1 | 0 | 1 | 1 | 7 |
| Wallace (2011) | 1 | 1 | 1 | 1 | 1 | 1 | 1 | 1 | 8 |
| Weiss (2013) | 1 | 1 | 1 | 1 | 1 | 1 | 1 | 1 | 8 |
| Wright (2011) | 1 | 1 | 1 | 1 | 0 | 1 | 1 | 1 | 7 |
| *Note.* Item 1: Is the hypothesis/aim/objective of the study clearly described?; Item 2: Are the main outcomes to be measured clearly described in the Introduction or Methods section?; Item 3: Are the characteristics of the patients included in the study clearly described?; Item 4: Are the main findings of the study clearly described?; Item 5: Were the subjects asked to participate in the study representative of the entire population from which they were recruited?; Item 6: Were those subjects who were prepared to participate representative of the entire population from which they were recruited?; Item 7: Were the statistical tests used to assess the main outcomes appropriate?; Item 8: Were the main outcome measures used accurate (valid and reliable)?; Yes = 1; No/unable to determine = 0. | | | | | | | | | |
